# Within-person reproducibility of proteoforms related to inflammation and renal dysfunction

**DOI:** 10.1038/s41598-022-11520-1

**Published:** 2022-05-06

**Authors:** Jie Gao, Adrian McCann, Johnny Laupsa-Borge, Ottar Nygård, Per Magne Ueland, Klaus Meyer

**Affiliations:** 1 Department of Clinical Science, Laboratory of Clinical Biochemistry, University of Bergen, Laboratory Building, 9th Floor, Jonas Lies veg 87, 5021 Bergen, Norway; 2 Bevital AS, Jonas Lies veg 87, 5021 Bergen, Norway; 3 Department of Heart Disease, Haukeland University Hospital, 5021 Bergen, Norway

**Keywords:** Biomarkers, Analytical chemistry

## Abstract

Protein biomarkers and microheterogeneity have attracted increasing attention in epidemiological and clinical research. Knowledge of within-person reproducibility over time is paramount to determine whether a single measurement accurately reflects an individual’s long-term exposure. Yet, research investigating within-person reproducibility for proteoforms is limited. We investigated the reproducibility of the inflammatory markers C-reactive protein (CRP), serum amyloid A (SAA), and calprotectin (S100A8/9), and the renal function marker cystatin C (CnC) using a novel immuno-MALDI-TOF MS assay. Reproducibility, expressed as intraclass correlation coefficient (ICC), was calculated for 16 proteoforms using plasma samples of the Western Norway B Vitamin Intervention Trial (WENBIT) cohort collected 1–3 y apart from 295 stable angina pectoris (SAP) patients and 16 weeks apart from 38 subjects of the Intervention with Omega Fatty Acids in High-risk Patients with Hypertriglyceridemic Waist (OMEGA) trial with abdominal obesity but no other documented co-morbidities. ICCs for inflammatory markers were lower in WENBIT (CRP: 0.51, SAAt: 0.38, S100At: 0.31) compared to OMEGA subjects (CRP: 0.71, SAAt: 0.73, S100At: 0.48), while comparable for CnCt (WENBIT: 0.69, OMEGA: 0.67). Excluding SAP patients with elevated inflammation (CRP > 10 µg/ml) increased the ICC of SAAt to 0.55. Reduction of the time interval from 3 to 1 y in WENBIT group increased ICCs for all proteoforms. With a few exceptions ICCs did not differ between proteoforms of the same biomarker. ICCs were highest in OMEGA subjects with fair-to-good reproducibility for all markers. Reproducibility of SAA and S100A8/9 proteoforms in the WENBIT cohort was related to inflammation. This work will inform future clinical and epidemiological research which relies on single time point biomarker assessment to investigate inflammation and renal function.

## Introduction

Knowledge of within-person reproducibility over time is crucial for the interpretation of data on biomarkers in epidemiological and clinical research based on a single measurement. Fluctuations unrelated to disease status will introduce regression dilution bias, and thereby attenuate the “true” association between exposure and disease risk^1^. Therefore, in prospective cohort studies relying on a single measurement, it is essential that the within-person variance in biomarker concentration is small in comparison to the between-person variance. The within-person reproducibility can be expressed as the ratio of between-person variation to the total variance and is defined as the intraclass correlation coefficient (ICC)^2^. The total variance is the sum of the within- and between person variance, including all variability related to pre-analytical sample handling and storage, technical measurements, and biological fluctuations^3^.

Protein biomarkers have attracted growing interest for the purpose of diagnosis, prognosis, and treatment monitoring of many diseases during the past decade^4,5^. Protein microheterogeneity largely caused by genetic polymorphisms, mutations, and posttranslational modifications (PTMs) has been related to different pathologies and may become an important feature of personalized medicine^6–9^. Various novel analytical technologies, many based on mass spectrometry, have been established for the detection of protein microheterogeneity and facilitate quantification of multiple biomarker proteoforms at high-throughput and low sample volume requirements^10–12^.

We investigated the within-person reproducibility of the inflammatory markers C-reactive protein (CRP), serum amyloid A (SAA), and calprotectin (S100A/9), and the renal function marker cystatin C (CnC) using a novel immuno-MALDI-TOF MS assay^13^. While ICCs have been reported earlier for the total protein concentrations of these markers, reproducibility of the 16 different proteoforms is unknown. ICCs of biomarkers may vary between cohorts (lifestyle, gender, clinical status, etc.) and study designs (size, duration, number of sampling intervals). Thus, we choose samples from two quite different studies, representing clinical patients and subjects with abdominal obesity but no other documented co-morbidities, to illustrate the potential for ICC variability of the four investigated biomarkers, which we think is especially important when comparing ICCs of the investigated protein biomarkers to those reported in the literature.

## Methods

### Study populations and sample collection

Within-person reproducibility over time was investigated in two different cohorts, Western Norway B Vitamin Intervention Trial (WENBIT) and Intervention With Omega Fatty Acids in High-risk Patients with Hypertriglyceridemic Waist (OMEGA), consisting of 295 stable angina pectoris (SAP) patients and 38 subjects with abdominal obesity but on other documented co-morbidities, respectively. The clinical characteristics of the participants have been summarized in Table 1 for both cohorts.

**Table 1:** Characteristics of participants of WENBIT and OMEGA cohorts.

Characteristics	WENBIT			OMEGA
	BL	1Y	END	BL/END
Sample size, n	295			38
Age, y (mean (SD))	61.3 (9.5)	62.3 (9.5)	64.3 (9.5)	55.6 (9.3)
**Gender (n (%))**				
Male	239 (81.5)			23 (59)
Female	56 (18.5)			15 (41)
**Clinical measurements (mean (SD))**				
BMI, kg/m^2^	27.3 (4.1)	–	–	29.2 (4.11)
Waist circumference, cm	98.7 (12.2)	–	–	104 (10.2)
eGFR, mL/min/1.73 m^2^*	88.1 (16.8)	87.2 (16.7)	85.1 (17.7)	91.1 (29.1)

*eGFR was calculated using the MDRD study equation as 186 × [serum creatinine (mg/dl)]^−1.154^ × (age)^−0.203^ × (0.742 if female).

Patients of the WENBIT cohort were randomly selected from the placebo control group of this study^14^, collected over a period of 3 years, and who suffered from stable angina pectoris (SAP) and had undergone coronary angiography for suspected coronary artery disease. All WENBIT participants provided written informed consent. The study protocol was in accordance with the principles of the Declaration of Helsinki and was approved by the Regional Committee for Medical and Health Research Ethics, the Norwegian Medicines Agency, and the Data Inspectorate. The ClinicalTrials.gov identifier was NCT00354081.

Blood samples of the OMEGA trial^15^ were collected over a period of 16 weeks. OMEGA subjects had participated in a crossover intervention study investigating the effects of omega-3 and omega-6 oil supplementation. Briefly, the study included volunteers who had increased waist circumference (≥ 94 cm in men, ≥ 80 cm in women) and were physical inactive (< 2 h vigorous/active exercise training per week). Samples taken at baseline (week 0) and after a wash-out phase at week 16 (7 weeks of the first intervention period plus 9 weeks wash-out phase) were utilized for the purpose of the present study. The OMEGA study was conducted according to the guidelines in the Declaration of Helsinki, and was approved by the Regional Committee for Medical and Health Research Ethics (2014/2336/REK South-East). The written informed consent was obtained from each participant before study.

All samples investigated were EDTA plasma samples, stored at -80 °C within 30 min after collection.

### Laboratory analyses

Samples were analyzed by a novel immuno-MALDI-TOF MS assay described previously^13^. Briefly, 20 µL EDTA plasma were spiked with 20 µL internal standards of polyhistidine-tagged recombinant proteins and incubated with antibody-immobilized paramagnetic beads. After intensive washing, proteins were eluted from the beads and analyzed by MALDI-TOF MS. Samples were processed in 96-microtiter plates using a Hamilton MircolabStar (Bonaduz, Switzerland) and CyBi-Disk robot from Analytik Jena (Jena, Germany).

### Nomenclature of proteoforms

SAAt, S100A8/9t and CnCt represented the total concentrations of the corresponding proteins. N-terminally truncated SAA, S100A8/9 and CnC were labelled with a “d” and the one-letter code(s) of the missing amino acids. SAA proteoforms were abbreviated according to the isoforms expressed by the *SAA1* or *SAA2* gene. Monomers of S100A8/9 were denoted as S100A8 and S100A9. S100A9tr was short for the shortest truncation of S100A9 which missed 5 amino acids. The native and the hydroxylated forms of CnC were abbreviated as CnCn and CnCo, respectively^16^.

### Statistical analyses

Age, body mass index (BMI), waist circumference and estimated glomerular filtration rate (eGFR) of the participants in both cohorts were indicated as arithmetic mean (SD). Protein and proteoform concentrations were presented as geometric means with 95% CIs. Proteoform concentrations were determined either as absolute levels or as values relative to the total concentration of the biomarker. Deviation of geometric mean concentrations between time points were determined by Student paired *t*-test. Correlation of biomarker concentrations between baseline (BL) and end of follow-up (END) were investigated by Spearman rank test. Within-person reproducibility was expressed as ICC and calculated using ln-transformed values and an ICC (1,1) model^17^. ICCs were classified according to Rosner as poor (< 0.4), fair-to-good (0.4–0.75), and excellent (≥ 0.75)^2^. Within- and between-person CVs were determined by calculating the square root of the variance components from the random-effect mixed model and were classified as high (> 100%), moderate (50–100%) and low (< 50%). The program R version 3.5.3 was used for statistical analyses, and the packages “DescTools”, “stat” and “ICC” (ICCest function) were used for geometric mean (95% CIs), Spearman rank test, Student *t*-test and ICC calculation, respectively.

## Results

### Total concentrations across time

**Figure 1:**
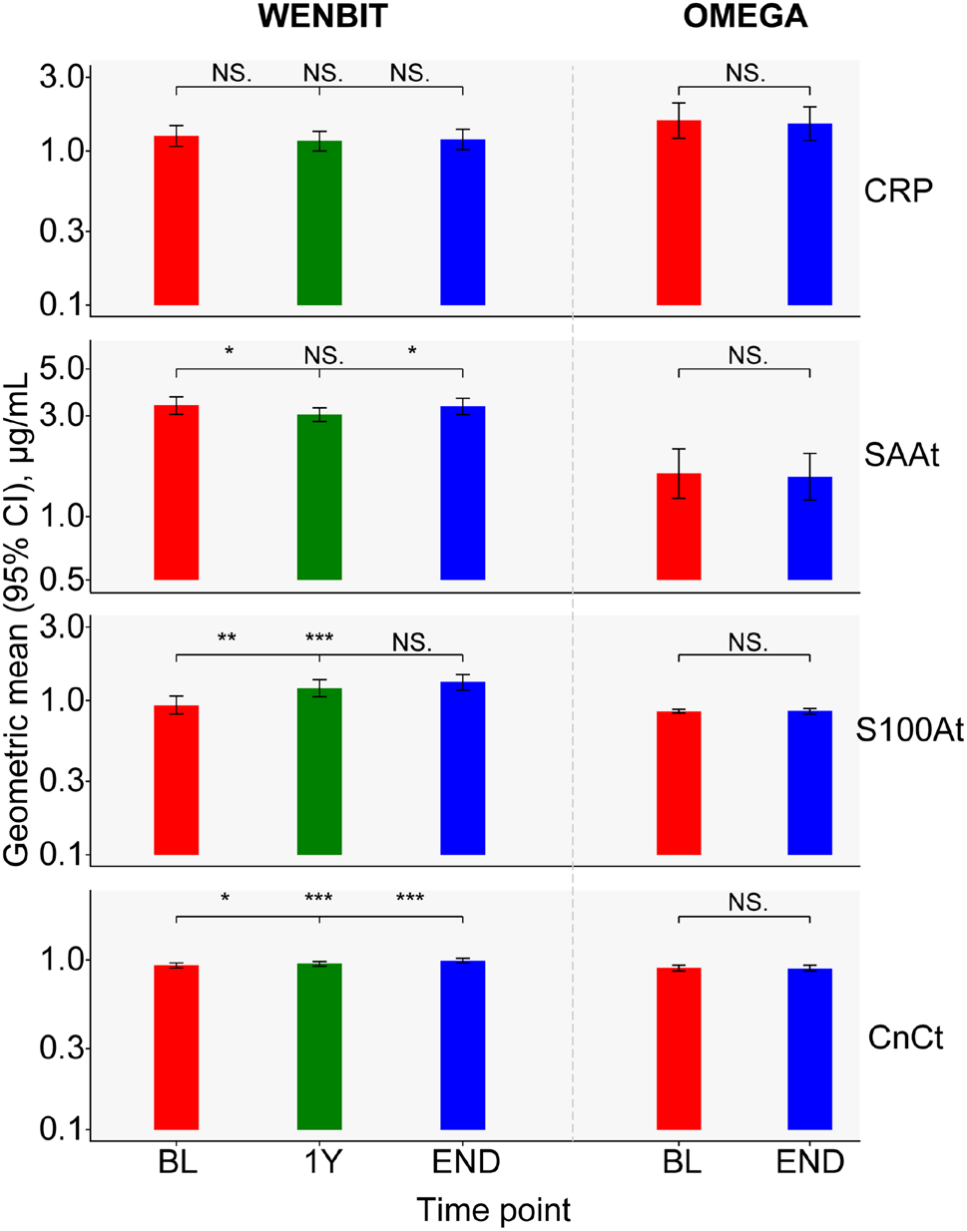
Total plasma concentrations of the four protein biomarkers in WENBIT and OMEGA samples. Samples were collected at 3 visits over 3y from 295 SAP patients enrolled in WENBIT and at 2 visits over 16 weeks from 38 participants in OMEGA. The concentrations were presented as geometric mean with 95% CIs, and the difference between concentrations for any two visits, investigated by *t*-test, were indicated in the order of BL versus 1Y, BL versus END, and 1Y versus END for WENBIT, and BL versus END for OMEGA. *NS* Not significant; *, *p* < 0.05; **, *p* < 0.01; ***, *p* < 0.001.

The total concentrations of the four protein biomarkers determined in the WENBIT and OMEGA cohorts are presented in Fig. 1. Levels of CRP and CnCt were comparable in both cohorts, while concentrations of SAAt and S100At were higher in WENBIT than OMEGA. Levels of CRP did not change during the follow up periods in WENBIT and OMEGA. Total plasma concentrations of SAA, S100A and CnC were stable in OMEGA, but changed in WENBIT. Plasma levels of S100At and CnCt increased significantly during the 3 years of follow up in WENBIT.

### Within- and between-person variability of biomarkers and proteoforms

The geometric mean (for all time points) for total protein and proteoform, Spearman correlation (BL vs END), within- and between-person coefficient of variation (CV), and ICC (95% CI) are shown for WENBIT (Table 2) and OMEGA (Table 3) participants.

**Table 2:** Concentrations and within-person reproducibility of biomarkers in EDTA plasma samples from WENBIT.

Biomarker	Geometric mean (95% CI), µg/mL			*P*^b,c^	*Rho*^c^	Within-person CV (%)	Between-person CV (%)	ICC^c^ (95% CI)
	BL^a^	1Y	END^a^					
C reactive protein	1.25 (1.07, 1.46)	1.16 (1.00, 1.34)	1.19 (1.02, 1.38)	0.51	0.5	152.66	157.55	0.51 (0.44, 0.57)
**Serum amyloid A**								
SAAt	3.36 (3.05, 3.69)	3.04 (2.82, 3.27)	3.32 (3.04, 3.63)	0.79	0.5	81.80	60.25	0.38 (0.31, 0.45)
SAA1.1	0.77 (0.69, 0.86)	0.67 (0.61, 0.73)	0.74 (0.67, 0.82)	0.46	0.52	91.74	78.85	0.44 (0.37, 0.51)
SAA1.1dr	0.38 (0.34, 0.42)	0.36 (0.33, 0.39)	0.38 (0.34, 0.42)	0.98	0.55	88.68	73.49	0.43 (0.36, 0.50)
SAA1.1drs	0.22 (0.20, 0.24)	0.20 (0.19, 0.22)	0.22 (0.20, 0.24)	0.95	0.4	91.06	63.57	0.37 (0.29, 0.44)
SAA1.2	0.33 (0.30, 0.36)	0.28 (0.26, 0.30)	0.32 (0.29, 0.35)	0.35	0.43	89.73	61.87	0.36 (0.29, 0.43)
SAA1.3	0.32 (0.29, 0.36)	0.28 (0.26, 0.31)	0.31 (0.28, 0.34)	0.59	0.46	88.68	65.04	0.38 (0.31, 0.46)
SAA2.1	0.38 (0.34, 0.42)	0.35 (0.32, 0.38)	0.38 (0.35, 0.42)	0.93	0.43	92.06	68.10	0.39 (0.32, 0.46)
SAA2.1dr	0.22 (0.20, 0.25)	0.20 (0.19, 0.22)	0.22 (0.20, 0.25)	0.79	0.39	100.37	60.56	0.32 (0.24, 0.39)
SAA2.1drs	0.15 (0.14, 0.17)	0.14 (0.13, 0.15)	0.15 (0.14, 0.17)	0.54	0.36	92.45	59.70	0.34 (0.26, 0.42)
**Calprotectin**								
S100At	0.93 (0.82, 1.07)	1.20 (1.06, 1.37)	1.32 (1.17, 1.49)	< 0.001	0.29	155.98	86.45	0.31 (0.23, 0.38)
S100A8	0.60 (0.52, 0.69)	0.79 (0.69, 0.90)	0.87 (0.76, 0.98)	< 0.001	0.29	165.79	94.42	0.32 (0.24, 0.39)
S100A9	0.09 (0.06, 0.13)	0.15 (0.12, 0.19)	0.18 (0.15, 0.21)	< 0.001	0.33	180.54	89.92	0.28 (0.21, 0.35)
S100A9dm	0.05 (0.04, 0.06)	0.06 (0.07, 0.07)	0.07 (0.06, 0.08)	< 0.001	0.34	143.31	78.57	0.30 (0.22, 0.37)
S100A9tr	0.12 (0.10, 0.13)	0.14 (0.13, 0.16)	0.16 (0.14, 0.18)	< 0.001	0.28	139.81	76.80	0.30 (0.22, 0.37)
**Cystatin C**								
CnCt	0.93 (0.90, 0.96)	0.95 (0.92, 0.98)	0.99 (0.96, 1.02)	< 0.001	0.62	15.70	24.56	0.69 (0.64, 0.74)
CnCn	0.30 (0.29, 0.31)	0.30 (0.29, 0.31)	0.31 (0.30, 0.32)	< 0.01	0.69	14.86	25.49	0.73 (0.68, 0.77)
CnCo	0.36 (0.35, 0.37)	0.36 (0.34, 0.37)	0.37 (0.36, 0.38)	< 0.05	0.67	14.57	24.96	0.73 (0.68, 0.77)
CnCds	0.09 (0.09, 0.10)	0.10 (0.10, 0.11)	0.11 (0.10, 0.11)	< 0.001	0.28	29.93	30.33	0.41 (0.34, 0.48)
CnCdssp	0.07 (0.06, 0.07)	0.07 (0.06, 0.07)	0.07 (0.06, 0.07)	0.84	0.44	32.55	26.65	0.51 (0.44, 0.57)

Samples collected at 3 time points over 3y apart from 295 SAP patients enrolled in the Western Norway B Vitamin Intervention Trial. Within- and between-person CVs were determined by taking the square root of the within- and between-person variance components from random-effects mixed model on the ln-transformed values.

^a^*BL* Baseline, *END* End of follow-up.

^b^Significant differences in biomarker geometric mean were evaluated by t-test.

^c^Values were obtained from the data at BL and END.

**Table 3:** Concentrations and within-person reproducibility of biomarkers in EDTA plasma samples from OMEGA.

Biomarker	Geometric mean (95% CI), µg/mL		*p*^b^	*Rho*	Within-person CV (%)	Between-person CV (%)	ICC (95% CI)
	BL^a^	END^a^					
C reactive protein	1.58 (1.21, 2.05)	1.50 (1.17, 1.93)	0.55	0.63	51.71	91.78	0.71 (0.51, 0.84)
**Serum amyloid A**							
SAAt	1.60 (1.22, 2.09)	1.54 (1.20, 1.99)	0.89	0.74	52.16	99.57	0.73 (0.54, 0.85)
SAA1.1	0.51 (0.38, 0.69)	0.50 (0.38, 0.66)	0.46	0.81	53.00	117.32	0.77 (0.60, 0.87)
SAA1.1dr	0.35 (0.26, 0.46)	0.32 (0.24, 0.43)	0.55	0.71	57.43	114.24	0.74 (0.55, 0.85)
SAA1.1drs	0.12 (0.09, 0.16)	0.11 (0.09, 0.15)	0.65	0.77	50.42	105.16	0.75 (0.58, 0.86)
SAA1.2	0.04 (0.03, 0.06)	0.03 (0.02, 0.05)	0.59	0.54	99.62	147.97	0.63 (0.40, 0.79)
SAA1.3	0.07 (0.05, 0.10)	0.07 (0.05, 0.10)	0.82	0.73	71.12	113.50	0.67 (0.44, 0.81)
SAA2.1	0.08 (0.06, 0.12)	0.09 (0.07, 0.11)	0.81	0.73	57.07	115.13	0.74 (0.56, 0.86)
SAA2.1dr	0.04 (0.03, 0.06)	0.04 (0.03, 0.06)	0.88	0.62	75.23	123.61	0.67 (0.46, 0.81)
SAA2.1drs	0.03 (0.02, 0.04)	0.03 (0.02, 0.04)	0.94	0.49	60.80	81.70	0.62 (0.38, 0.78)
**Calprotectin**							
S100At	0.85 (0.83, 0.88)	0.86 (0.82, 0.89)	0.25	0.58	7.94	7.65	0.48 (0.20, 0.69)
S100A8	0.51 (0.48, 0.55)	0.51 0.47, 0.56)	0.97	0.54	17.30	18.82	0.54 (0.28, 0.73)
S100A9	0.12 (0.09, 0.15)	0.11 (0.09, 0.15)	0.98	0.31	78.28	74.54	0.48 (0.20, 0.69)
S100A9dm	0.06 (0.05, 0.08)	0.07 (0.06, 0.08)	0.44	0.29	62.18	43.09	0.35 (0.05, 0.60)
S100A9tr	0.11 (0.10, 0.12)	0.11 (0.10, 0.12)	0.25	0.30	20.50	15.21	0.37 (0.07, 0.61)
**Cystatin C**							
CnCt	0.90 (0.86, 0.93)	0.89 (0.86, 0.93)	0.93	0.63	6.29	9.17	0.67 (0.46, 0.82)
CnCn	0.32 (0.31, 0.34)	0.33 (0.31, 0.34)	0.61	0.65	7.08	10.20	0.67 (0.45, 0.81)
CnCo	0.38 (0.37, 0.40)	0.38 (0.36, 0.39)	0.25	0.65	6.78	10.48	0.70 (0.49, 0.83)
CnCds	0.06 (0.06, 0.07)	0.06 (0.06, 0.07)	0.98	0.39	17.27	20.53	0.58 (0.33, 0.75)
CnCdssp	0.06 (0.05, 0.06)	0.06 (0.05, 0.06)	0.34	0.63	15.61	21.56	0.64 (0.42, 0.79)

Samples collected at 2 time points over 16 weeks apart from 38 subjects in the OMEGA Trial. Within- and between-person CVs were determined by taking the square root of the within- and between-person variance components from random-effects mixed model on the ln-transformed scale.

^a^*BL* Baseline, *END* End of follow-up.

^b^Significant differences in biomarker geometric mean were evaluated by t-test.

In accordance with the total biomarker concentrations, plasma levels of S100A8/9 and most CnC proteoforms increased significantly over time in the WENBIT group (Table 2). Within-person CVs were high for CRP and S100A8/9, moderate for SAA, and low for CnC. Similar CVs were obtained for between-person variation, with the exception of S100A8/9, which demonstrated moderate variation.

For OMEGA, plasma levels of proteoforms did not differ significantly between baseline and end (Table 3). Within-person CVs were moderate for CRP and SAA, and low for CnC. Variance differed between S100A8/9 proteoforms and ranged from moderate (S100A9, S100A9dm) to low (S100A8, S100A9tr). Between-person CVs of S100A8/9 and CnC were similar to their within-person variation, whereas between-person CVs for CRP and SAA were higher.

### Within-person reproducibility of biomarkers and proteoforms

In WENBIT (Table 2), the ICCs of CRP and CnC were highest among the protein biomarkers, and ranged between 0.41 and 0.73, while ICCs of SAA and S100A8/9 were lower, ranging from 0.28 to 0.44. ICCs were slightly higher than Spearman’s Rho for CRP, S100A8/9, and CnC, and lower for SAA. Reproducibility of CRP, SAA and CnC in OMEGA (Table 3) were similar and ICCs ranged from 0.58 to 0.77. ICCs of S100A8/9 was lower and varied from 0.35 to 0.54. ICCs were higher than Spearman’s Rho for CRP and CnC, and comparable for SAA and S100A8/9. However, due to the low number of subjects in the OMEGA trial, 95% CIs for ICCs were 2–3 times larger than those observed in WENBIT.

**Figure 2:**
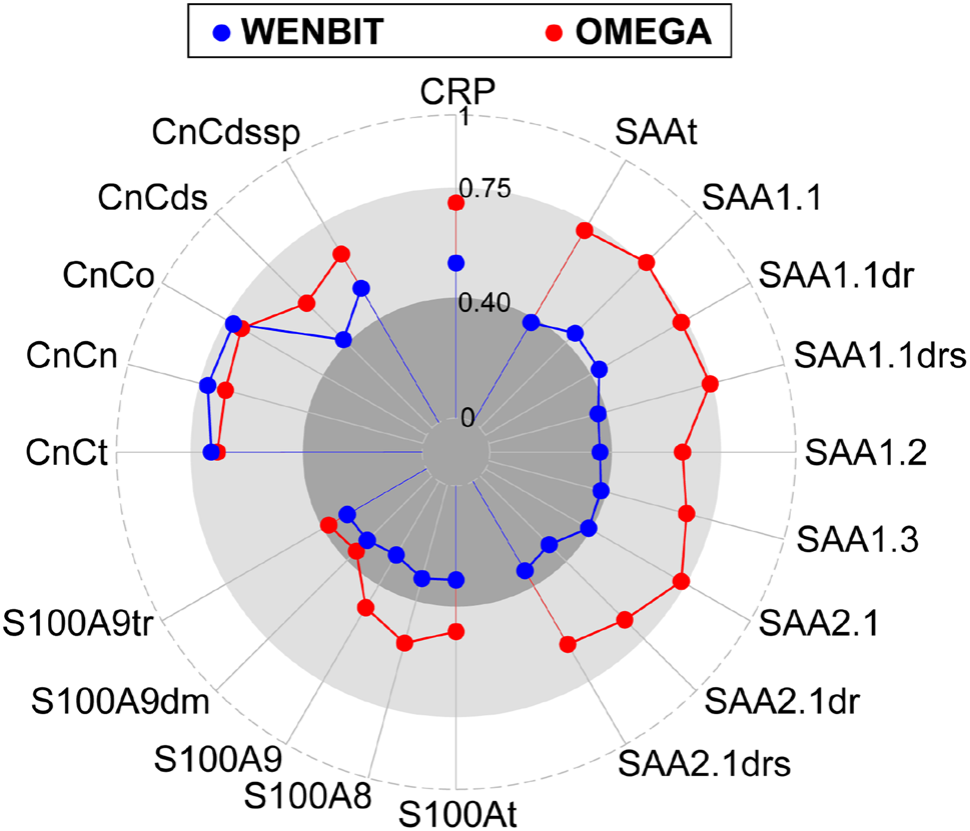
Comparison of intraclass correlation coefficients (ICCs) in WENBIT and OMEGA. ICCs were calculated using ln-transformed analyte values. Higher ICCs were obtained for CRP, SAA, S100A8/9 and the proteoforms in OMEGA than WENBIT while similar reproducibility was obtained for CnC and its proteoforms. Data were taken from Tables 2 and 3. Thresholds of ICCs (< 0.4: poor; 0.4–0.75: fair to good; > 0.75: excellent) were marked by different tones of grey.

ICCs of total protein and proteoform concentrations were compared between cohorts and illustrated as radar plots (Fig. 2). In WENBIT, ICCs ranged from fair-to-good for CRP, and poor for SAA and S100A8/9. In contrast, fair-to-good reproducibility was observed for most proteoforms in OMEGA, with ICCs for CRP and SAA close to excellent. SAA1.1 in the OMEGA cohort was the only proteoform demonstrating excellent reproducibility. ICCs of CnC were fair-to-good and showed comparable values in both study groups. Notably, differences in ICCs between proteoforms of the same biomarker were generally small with exception of a few low-abundance proteoforms.

**Figure 3:**
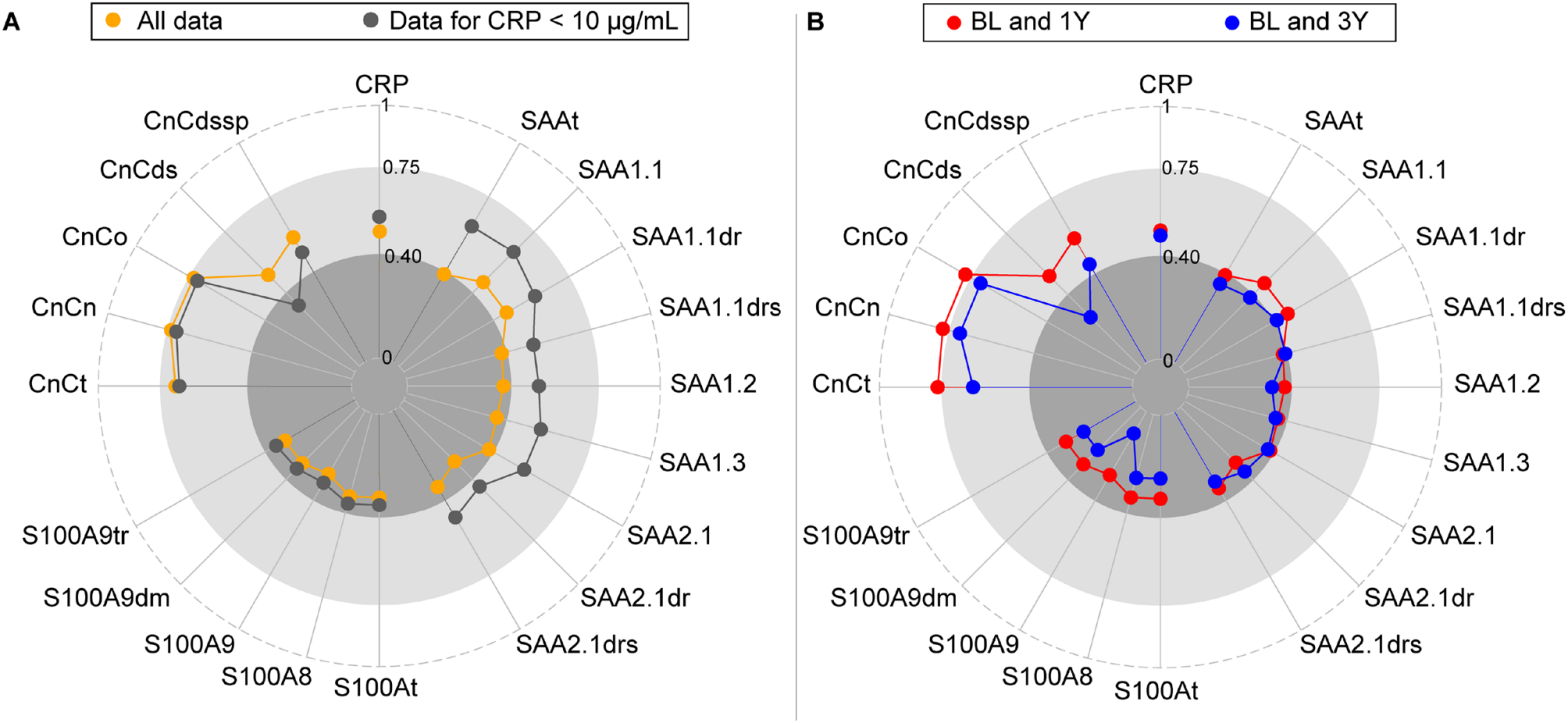
ICC changes after excluding outliers and according to the time interval from 1 to 3y. (**A**) After excluding 29 subjects with CRP > 10 µg/mL (outliers), ICCs of SAA increased from poor to fair-to-good while ICCs for the other biomarkers changed slightly. (**B**) ICCs of all the analytes increased across the time span. Thresholds of ICCs (< 0.4: poor; 0.4–0.75: fair to good; > 0.75: excellent) were marked by different tones of grey.

Additional sub-group analyses were performed in the WENBIT cohort. The impact of acute inflammation on ICCs was investigated by excluding 29 patients with CRP > 10 µg/ml (Fig. 3A). Removal of those with elevated CRP (reflective of increased acute systemic inflammation) improved the ICCs of all inflammatory markers. While the increase was marginal for CRP and S100A8/9, ICCs of SAA proteoforms increased markedly and changed performance from poor to fair-to-good. Furthermore, ICCs were calculated for the different time intervals between baseline and 1 or 3 years (Fig. 3B). While ICCs for CRP and SAA were similar for both time intervals, reproducibility for S100A8/9 and CnC proteoforms were highest after 1 year follow up. Notably, the reproducibility of CnCn and CnCo were excellent at 1 year, fair-to-good at 3 years.

### Variability of proteoform distributions across observation period

**Figure 4:**
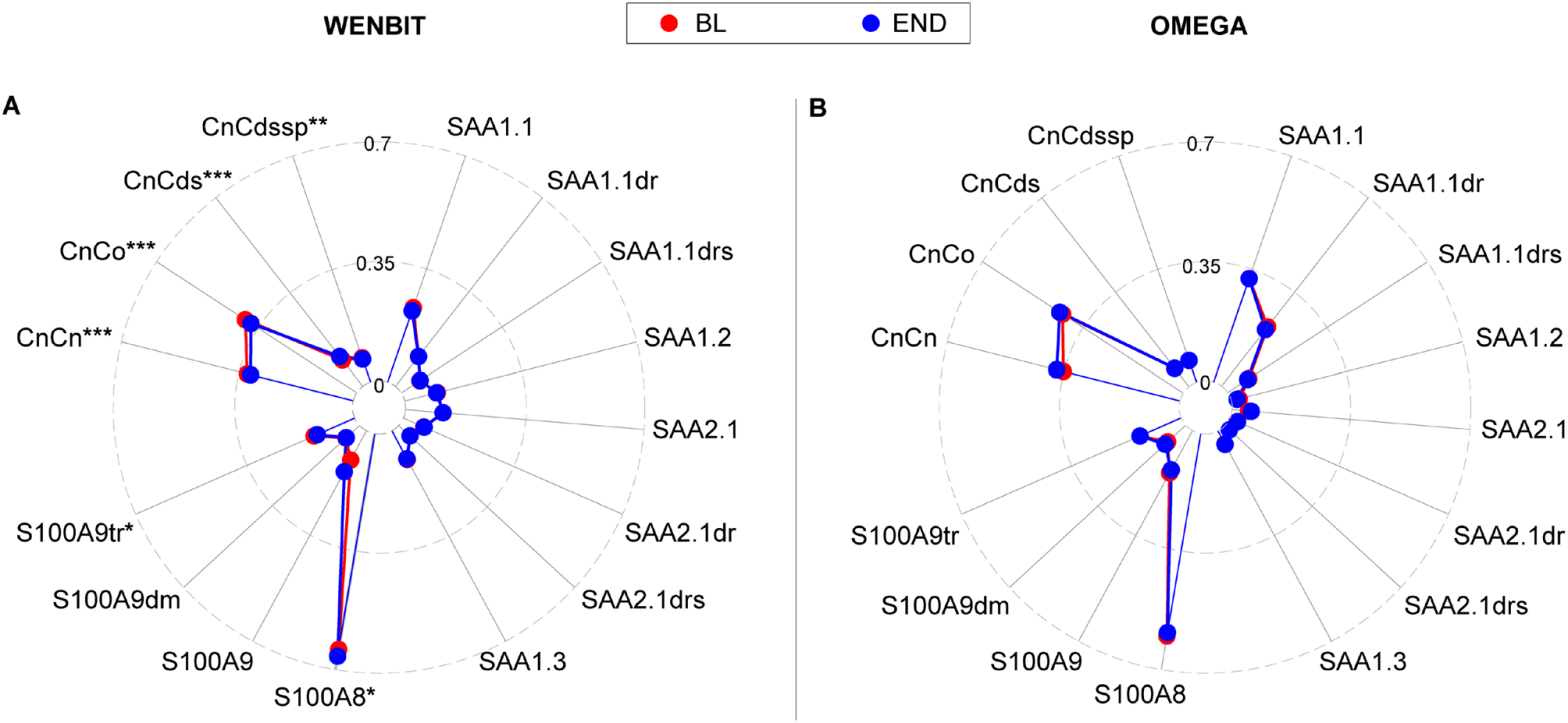
Comparison of proteoform distributions in WENBIT (**A**) and OMEGA (**B**). Values are geometric mean of relative proteoform concentrations, i.e. fraction of the total concentration of the actual biomarker. Distributions of SAA, S100A8/9 and CnC proteoform levels were highly stable over time in both groups. Small significant differences were observed for CnC and S100A8/9 in WENBIT. *BL* Baseline, *END* End of follow-up. *, *p* < 0.05; **, *p* < 0.01; ***, *p* < 0.001.

The variability of proteoform distributions was investigated in both cohorts by comparing the geometric means of relative proteoform concentrations (Fig. 4) and the within- and between-person variances based on both absolute and relative levels (Supplemental Fig. 1). Relative levels of SAA, S100A8/9 and CnC proteoforms were stable over time in both cohorts. However, weak but significant differences (*p* < 0.05) were observed for S100A8/9 and CnC in WENBIT. In addition, within- and between-person variances of relative values were generally lower than for absolute proteoform concentrations (Supplemental Fig. 1), and ranged between 5–52% and 3–82% in WENBIT and OMEGA, respectively. Lowest variation was observed for the proteoforms of CnC with an average CV of 10%.

## Discussion

### Biomarker levels and within-person reproducibility

We determined circulating concentrations and ICCs of the inflammatory markers CRP, SAA, and S100A8/9, and the renal function marker CnC in two different cohorts. The plasma concentrations and within-person reproducibility for these protein biomarkers differed between the two cohorts. Plasma levels of SAAt and S100At were higher in WENBIT compared to OMEGA subjects, reflecting prevalent inflammation likely related to established CAD among the WENBIT participants^18,19^. Levels of CnCt were similar in both groups reflecting comparable renal function. ICCs for CRP, SAA and S100A8/9 were highest among the OMEGA subjects generally demonstrating fair-to-good within-person reproducibility. The lowest ICCs were observed for SAA and S100A8/9 in the WENBIT cohort. Within-person reproducibility of CnC proteoforms was fair-to-good and similar in both cohorts. Differences in ICCs between proteoforms of the same biomarker were generally small.

### Impact of inflammation, aging and time span on within-subject reproducibility in WENBIT

Within-person reproducibility for the four protein biomarkers and their proteoforms in the WENBIT group was related to inflammation and time span. Removal of CRP values > 10 µg/ml, indicating elevated systemic inflammation^20^, improved ICCs for all three inflammatory markers. Although CRP and SAA are both stimulated by IL-6 and highly correlated during inflammation^21^, the ICC improvement was more pronounced for SAA than CRP. This may be related to SAA’s role as an acute-phase protein with more pronounced elevation than CRP in response to inflammatory stimuli^22^. Reducing the time interval from 3 to 1 year increased the ICCs of S100A8/9 and CnC, with a concurrent, significant increase in concentrations of both biomarkers over longer-term follow-up. Aging-related elevation of S100At and CnCt levels has recently been associated with chronic inflammation and impaired renal function, respectively^23,24^. Our data suggested that ICC may be impacted by aging related changes such as declining renal function.

### Comparison with published data on reproducibility

Others have investigated within-person reproducibility of the selected protein biomarkers, but to our knowledge, this is the first publication to report on proteoform reproducibility. Comparison with other studies is difficult since models for ICC estimations were occasionally not defined and cohorts varied by sample size and time intervals. Several studies evaluating CRP variability, over short (weeks) and long term (≥ 3 y) with sample sizes of dozens to thousands, have reported fair-to-good and excellent reproducibility (0.61 < ICC < 0.77) for subjects with CRP values within the normal range (< 10 µg/mL)^25–28^, which are comparable with the results presented in the present paper. Highest reproducibility was obtained in a short-term study with a 2.5 weeks’ time interval^29^. A few studies have determined the ICC for SAAt. A large cohort consisting of 7000 healthy participants determined a value of 0.67 for SAAt, similar to the ICC for the WENBIT group after excluding subjects with CRP > 10 µg/mL^30^. Another study^31^, comparable to OMEGA in size and time interval, investigated 24 healthy participants over 5 weeks and reported a higher ICC of 0.85 for SAAt. Within-person reproducibility of S100At has been reported only once before in a study investigating 207 healthy subjects over a 4-month period^32^. Reproducibility was poor with an ICC of 0.38 but comparable with the value obtained for the WENBIT cohort after 1 year in the present study. The reproducibility of CnCt has been investigated in two small studies, consisting of 10 and 12 healthy individuals^33,34^. In contrast to our study, the reported ICCs were either excellent (ICC = 0.89) or poor (ICC = 0.27), which may be related to the different time spans of 1 and 102 weeks, respectively.

### Limitations

Sufficient sample sizes are required for precise estimation of ICCs. The sample sizes of WENBIT and OMEGA differed considerably, and 95% CIs varied between 5–26% and 13–86%, respectively. Smaller 95% CIs could have been achieved in OMEGA by repetition of duplicate or triplicate analyses for each time point^35^, but limited sample volume meant only one measurement could be performed. Also, within each cohort 95% CIs of ICCs differed strongly between biomarkers. Relative variation of 95% CIs increases with decreasing ICC, but decrease with increasing sample size. In order to achieve levels of precision for S100At similar to that of CnCt in the WENBIT cohort, sample size would have to be increased to approximately 3000 subjects^36^. A further limitation, includes the analyses of WENBIT and OMEGA samples on different days. Thus, potential effects of preanalytical factors or inter-day variation of the assay on biomarker concentrations cannot be excluded. However, control samples were used in each batch to control for variation. Finally, proportion of male vs female participants in WENBIT was imbalanced (81% vs 19%), but the impact of gender on ICC estimation is expected to be small compared to other factors.

## Conclusion

We investigated the within-person reproducibility of the inflammatory biomarkers C-reactive protein (CRP), serum amyloid A (SAA), and calprotectin (S100A8/9), and the renal function marker cystatin C (CnC) and their 16 different proteoforms. Within-person reproducibility was highest in the OMEGA trial with fair-to-good reproducibility for all four markers. ICCs of SAA and S100A8/9 in WENBIT appeared to be impacted by the underlying inflammation profile of the cohort. Proteoforms of the same marker demonstrated comparable reproduciblility, and proteoform distributions were highly consistent over time, although ICCs for S100A8/9 and CnC changed with time. This may be linked to a number of factors such as renal function. The presented within-person reproducibility data will help inform future epidemiological and clinical studies which include these protein biomarkers and allow for correction of potential regression dilution which impacts risk estimations’^1^.

## Supplementary Information

Supplementary Information.
