# Supplementary material for: Within-person reproducibility of proteoforms related to inflammation and renal dysfunction
Source: Sci Rep. 2022 May 6;12:7426. doi: 10.1038/s41598-022-11520-1 (PMC9076635; doi:10.1038/s41598-022-11520-1)

**Supporting information for:**

Within-person reproducibility of proteoforms related to inflammation and renal dysfunction

Jie Gao^1*^, Adrian McCann^2^, Johnny Laupsa-Borge^1^, Ottar Nygård^1,3^, Per Magne Ueland^2^, and Klaus Meyer^2^

^1^Department of Clinical Science, University of Bergen, and Laboratory of Clinical Biochemistry,

Haukeland University Hospital, 5021 Bergen, Norway

^2^Bevital AS, Jonas Lies veg 87, 5021 Bergen, Norway

^3^Department of Heart Disease, Haukeland University Hospital, 5021 Bergen, Norway

* Address correspondence to this author at: Laboratory building, 9^th^ floor, Jonas Lies veg 87, 5021 Bergen, Norway. Tel: +47-55-974697. Fax: +47-55-974605. Email: [Jie.Gao@uib.no](mailto:Jie.Gao@uib.no)


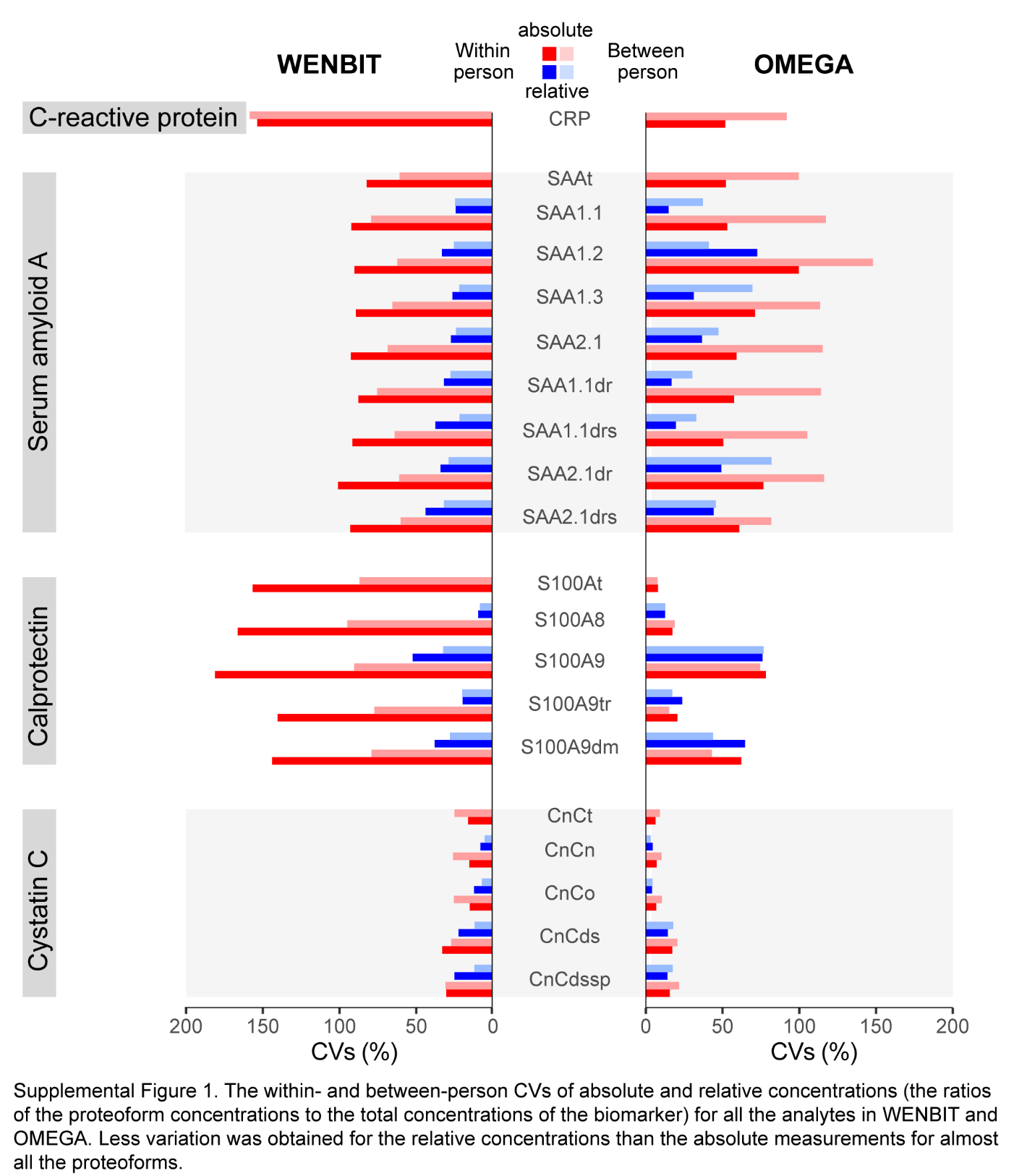

Supplement: Supplementary file 1 — Supplementary Information. [file 41598_2022_11520_MOESM1_ESM.docx]
